# Cognitive processes that indirectly affect olfactory dysfunction in Parkinson's disease

**DOI:** 10.1016/j.prdoa.2019.07.003

**Published:** 2019-07-20

**Authors:** Attakias T. Mertens, Jonathan B. Santo, Katerina Markopoulou, Bruce A. Chase

**Affiliations:** a Department of Psychology, University of Nebraska at Omaha, Omaha, NE, United States of America; b Department of Neurology, NorthShore University Health System, Evanston, IL, United States of America; c Department of Neurology, University of Chicago, Pritzker School of Medicine, Chicago, IL, United States of America; d Department of Biology, University of Nebraska at Omaha, Omaha, NE, United States of America

**Keywords:** Parkinson's disease, Olfaction, Memory, Cognition, Structural equation modeling, Decision making

## Abstract

**Introduction:**

Accurate early diagnosis of Parkinson's disease is hampered by its long prodromal period and the variable manifestations of its motor symptoms. While olfactory dysfunction can occur before motor-symptom onset and serve as a non-disease-specific diagnostic aid, its underlying causes are incompletely understood.

**Methods:**

Correlation analyses, univariate density estimates, ANOVA and regression evaluated relationships between scores on the Montreal Cognitive Assessment and Hopkins Verbal Learning Test and those on the University of Pennsylvania Smell Identification Test in 1280 Parkinson's Progression Markers Initiative subjects placed into five diagnostic categories. Structural equation modeling identified cognitive measures having significant indirect effects on olfactory-function-test scores.

**Results:**

Global cognition, verbal learning and memory, attention, delayed-recall, and visuospatial/executive function scores show weak-to-moderate, significant associations with olfactory-function-test scores. Associations are stronger in symptomatic than asymptomatic subjects having mutations in *LRRK2*, *GBA* or *SNCA*. Score distributions are nonuniform across diagnostic categories. Linear regression found that all cognitive measures except attention predicted olfactory-function-test scores. Three structural equation models assessing indirect effects of verbal learning/memory with either global cognition, visuospatial/executive function, or delayed-recall had a good statistical fit to the data. Only verbal learning/memory scores significantly help explain olfactory-function-test scores in all symptomatic diagnostic categories (−0.56 < b < −0.23, 0.001 < *P* < .005). Visuospatial/executive-function test scores help explain olfactory-function-test scores in both genetic Parkinson's disease diagnostic categories (−0.25 < b < −0.17, 0.032 < *P* < .033).

**Conclusion:**

Impaired verbal learning/memory and visuospatial/executive function contributes to lower performance on olfactory function tests in Parkinson's disease. As both of these domains impact decision-making, decision-making in turn may impact olfactory assessment in Parkinson's disease.

## Highlights

• Olfactory function is impaired in PD but the underlying mechanisms are unclear.
• Verbal learning/memory and visuospatial/executive function impact olfaction scores.
• Impaired decision making may contribute to low scores on olfaction tests in PD.

## Graphical abstract

**Figure:**
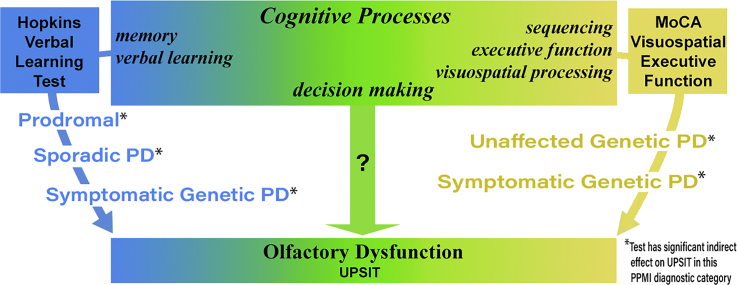

## 1 Introduction

Accurate diagnosis of Parkinson's disease (PD) is complicated by the variable manifestations of motor and non-motor symptoms. Assessment of olfactory dysfunction, an early non-motor symptom present in up to 90% of PD patients [1], may serve as an ancillary diagnostic criterion [[2], [3], [4], [5]]. However, it is not specific to PD: it is found in other neurodegenerative diseases and healthy aging [6,7]. It is therefore important to understand how its onset and progression relates to PD motor and non-motor manifestations.

Interpreting olfactory stimuli requires primary sensory detection along with multiple levels of associative sensory-signal processing. Clinically used tests of olfactory function implicitly measure multiple domains, as tests of odor detection, discrimination, and identification require information processing within the olfactory bulb and higher-order associative centers. They may be sensitive to specific types of cognitive impairment, such as memory decline that affects odor-memory retention or the associative processing of an odor memory.

If a subtle cognitive deficit contributes to lower scores on olfactory function tests, then subjects retaining some olfactory function might obtain similar scores but misidentify different odors upon retesting. Such odor-identification irreproducibility was observed but could not be explained by global-cognition test scores [8]. This suggests that routinely used clinical measures of cognitive performance in PD may have limited sensitivity to detect the cognitive deficits that impact performance on olfactory-function tests.

Here, we identify specific cognitive deficits that help explain lower scores on tests of olfactory function in PD and quantify the magnitude of their indirect effects by developing structural equation models (SEM) using data from the Parkinson's Progression Marker Initiative (PPMI). This report extends previous research demonstrating that memory is strongly related to olfaction [[9], [10], [11]] and builds on previously established correlative associations and predictive relationships between deficits in olfaction and verbal learning/memory in PD [12,13]. In addition to quantifying the contributions of verbal learning/memory, we also assess visuospatial/executive function, which is frequently impaired in PD [14], attention, and delayed-recall. Quantifying how poorer performance on tests of these cognitive measures contributes to lower scores on olfactory function tests provides insight into how and why olfactory test scores decline in some PD patients, and how potentially subtle alterations in cognition in PD impact seemingly unrelated clinical assessments.

## 2 Subjects and methods

### 2.1 Assessments

Data used in the preparation of this article were obtained from the PPMI database (www.ppmi-info.org/data) in September 2018. For study information, visit www.ppmi-info.org. We analyzed scores on the University of Pennsylvania Smell Identification Test (UPSIT), a forced-choice test of odor identification [15], the Hopkins Verbal Learning Test (HVLT), an assessment of verbal learning and memory that is a significant predictor of dementia [16,17], and the Montreal Cognitive Assessment (MoCA), which evaluates visuospatial/executive function, attention/concentration, naming, language, immediate and delayed-recall, calculations and orientation, and accurately assesses cognitive impairment in PD [14,18]. We used only the three free-recall memory trials of the HVLT, since they and the UPSIT similarly rely on the processes of short-term/working memory, semantic and/or episodic memory, and association. They may be used in the UPSIT to retain an odor memory while selecting among words for an olfactory cue, while they are used in the free-recall trials of the HVLT to recall auditory cues (words read aloud). We also assessed MoCA subscores for visuospatial/executive function (Visu-Exec), attention/concentration (Attention), and delayed-recall (Delayed-Recall), as these cognitive domains are commonly and potentially differentially affected in PD and are also used in olfactory coding and memory [[19], [20], [21]]. These subscores have utility in understanding cognitive phenotypes of PD [14], akin to using Unified Parkinson's Disease Rating Scale subscores in assessing motor subtypes. Since the UPSIT, HVLT, and MoCA were administered together only at study baseline, we analyzed only baseline assessments.

### 2.2 Subjects

We analyzed data from five well-defined PPMI-assigned diagnostic categories (https://www.ppmi-info.org/study-design/). The main diagnostic categories were sporadic PD (≥2 of resting tremor, bradykinesia, or rigidity, with resting tremor or bradykinesia required, or either asymmetric resting tremor or asymmetric bradykinesia; PD diagnosis ≤2 years; Hoehn and Yahr (H&Y) I-II; scan-confirmed dopaminergic deficit; ≥30 years at diagnosis; no dopaminergic medications ≥6 months after baseline assessment) and healthy controls (≥30 years, no active neurological disorder or first-degree relative with idiopathic PD, MoCA ≥26, scan-negative for dopaminergic deficit). We included two diagnostic categories of subjects who are at increased genetic-risk of developing PD, symptomatic-genetic-PD (motor symptoms in sporadic-PD diagnostic category, PD diagnosis ≤7 years, H&Y I-III, ≥18 years, and a *LRRK2*, *GBA*, or *SNCA* mutation) and asymptomatic-genetic-PD (no PD diagnosis at baseline, ≥45 years, mutation or first-degree relative with mutation in either *LRRK2* or *GBA*, or ≥ 30 years, mutation or first-degree relative with mutation in *SNCA*), as the disease phenotypes are similar to sporadic PD and their inclusion allows for comparisons in individuals having a known disease cause before and after motor-symptom onset. We included the possible-prodromal-PD diagnostic category (≥60 years, hyposmia and/or REM-behavior sleep disorder (RBD)), as this is a potentially informative comparison group due to a high prevalence of hyposmia (71% have UPSIT < 21).

### 2.3 ANOVA and SEM

Since MoCA, Visu-Exec, and UPSIT scores were somewhat skewed, we used the robust maximum likelihood parameter estimator [22]. ANOVA testing for homogeneity of variance revealed some violation (1.3:1 male:female). ANOVA *t* and *F* statistics are sufficiently robust to handle this violation [23].

We used SEM (Mplus version 7.11), which incorporates both factor and regression analyses [24,25], to evaluate models in which cognitive processes are hypothesized to have indirect effects on (*i.e.*, help explain) the relationship between olfactory dysfunction and a PPMI-diagnostic category, relative to healthy controls. Significant models with a good fit to the data had model chi-square (χ^2^) α ≥ 0.05, comparative fit index (CFI) ≥ 0.93 (1 = perfect fit), root mean square error of approximation (RMSEA) ≤0.08, and standard root mean square residual (SRMR) ≤0.08 [24]. We used such models to infer whether a cognitive measure has an indirect effect on, or a significant role in explaining, the relationship between UPSIT scores and a diagnostic category, and to infer its magnitude.

## 3 Results

### 3.1 Descriptive statistics and correlations

**Fig. 1:**
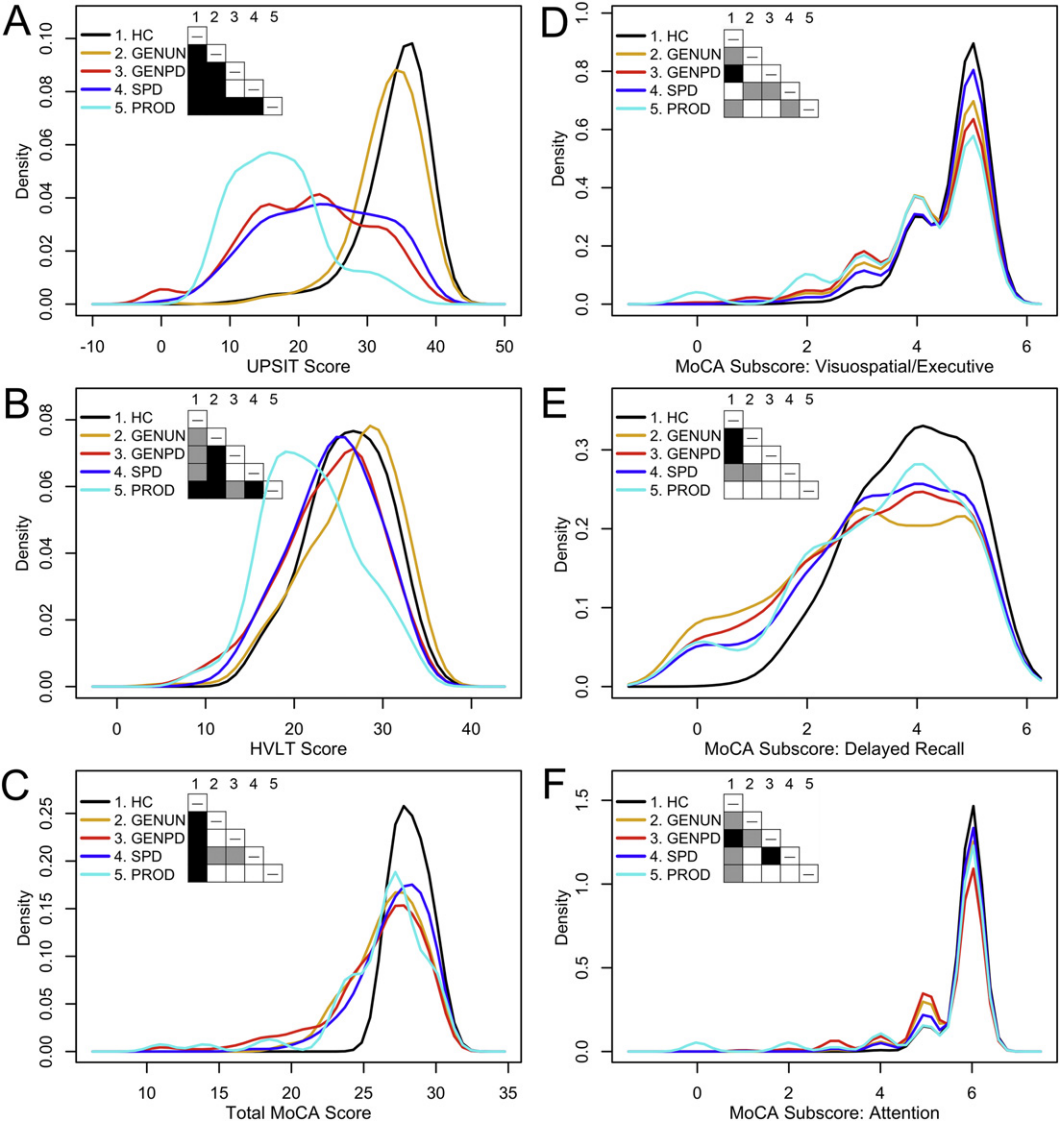
Graphical comparison of univariate density estimates for study variables. (A) UPSIT score. (B) HVLT score. (C) MoCA score. (D) Visuospatial/Executive Subscore. (E) Delayed-Recall Subscore. (F) Attention Subscore. Table cells within each panel are shaded to indicate the *P* value (white: *P* ≥ .05, gray: 0.05 > *P* ≥ .001, black: *P* < .001) obtained from pairwise nonparametric bootstrap tests of equal densities using 1000 permutations [26]. PPMI-defined diagnostic categories: HC = healthy controls; GENUN = individuals with asymptomatic genetic (*LRRK2*, *SNCA*, or *GBA*) PD; GENPD = individuals with symptomatic genetic PD; SPD = individuals with sporadic PD at baseline; PROD (possible prodromal PD) = individuals diagnosed with hyposmia and/or RBD.

Table 1 presents descriptive statistics for study subjects and Pearson zero-order correlations between study variables. The latter reveal weak-to-moderate, significant positive associations between scores on tests of olfaction and cognition: if a subject had lower scores on one test, they tended to score lower on the others as well. In contrast, scores showed weak negative correlations with age. To understand whether positive correlations between scores reflect similar score distributions in all diagnostic categories, we compared their distributions and univariate density estimates [26] between diagnostic categories. Score distributions are nonuniform between diagnostic categories (Fig. 1, Supplemental Fig. 1, Supplemental Fig. 2). Specifically, the distributions of UPSIT, HVLT, and MoCA scores in healthy controls are clearly distinct from those in other diagnostic categories. Therefore, we asked how the study measures were correlated for each of the diagnostic categories compared to healthy controls (Supplemental Table 1). This confirmed previously demonstrated positive associations found in idiopathic PD between scores on the USPIT and tests of verbal learning/memory [12,13], and discovered significant associations between scores on the UPSIT, MoCA, HVLT, Visu-Exec and Delayed-Recall in each symptomatic diagnostic category. Strikingly, these associations are stronger in symptomatic than asymptomatic subjects with genetic-PD. Taken together, these analyses suggest that the underlying neurodegenerative process(es) affect(s) interactions between the tested cognitive domains.

### 3.2 ANOVA

Separate, univariate ANOVAs investigated whether mean UPSIT, HVLT, MoCA, Visu-Exec, Delayed-Recall and Attention scores differ between the five diagnostic categories, holding age and sex constant as covariates. There are significant differences between the effects of diagnostic categories on scores on the UPSIT (*F*_6,1273_ = 138.63, *P* = 3.19 × 10^−135^, η^2^ = 0.395), HVLT (*F*_6,1273_ = 31.68, *P* = 1.20 × 10^−35^, η^2^ = 0.126), and MoCA (*F*_6,1269_ = 26.23, *P* = 1.56 × 10^−29^, η^2^ = 0.011), and on MoCA subscores for Visu-Exec (*F*_6,1273_ = 9.78, *P* = 1.47 × 10^−10^, η^2^ = 0.044), Delayed-Recall (*F*_6,1273_ = 23.21, *P* = 5.54 × 10^−13^, η^2^ = 0.049) and Attention (*F*_6,1273_ = 9.245, *P* = 6.18 × 10^−10^, η^2^ = 0.037).

**Table 1:** Descriptive statistics of study subjects and correlations between study variables.

Study variable	Mean ± standard deviation					Pearson correlation coefficient and *P*-value^b^						
	PPMI diagnostic category					Study variable						
	Healthy controls n = 198 F = 71	Asymptomatic genetic Parkinson's disease n = 310 F = 187	Symptomatic genetic Parkinson's disease n = 220 F = 113	Sporadic Parkinson's disease n = 491 F = 169	Possible prodromal Parkinson's disease n = 61 F = 13	UPSIT n = 1280	HVLT n = 1280	MoCA n = 1276	Visu-Exec n = 1280	Delayed recall n = 1279	Attention n = 1280	Age n = 1280
UPSIT (40)^a^	34.0 ± 4.8	33.0 ± 4.9	21.5 ± 8.9	23.4 ± 8.7	17.2 ± 6.6	–						
HVLT (30)^a^	26.0 ± 4.5	26.5 ± 5.2	24.0 ± 5.6	24.4 ± 4.9	22.0 ± 5.3	**0.281** 1.2 × 10^−24^	–					
MoCA (30)^a^	28.2 ± 1.1	26.7 ± 2.3	25.9 ± 3.5	27.1 ± 2.3	26.2 ± 3.6	**0.182** 6.2 × 10^−11^	**0.449** 1.9 × 10^−64^	–				
Visu-Exec (5)^a^	4.66 ± 0.59	4.35 ± 0.88	4.22 ± 0.99	4.49 ± 0.80	4.03 ± 1.21	**0.132** 2.2 × 10^−6^	**0.195** 1.9 × 10^−12^	**0.353** 7.8 × 10^−39^	–			
Delayed-Recall (5)^a^	3.88 ± 0.98	3.04 ± 1.57	3.22 ± 1.48	3.35 ± 1.41	3.31 ± 1.43	**0.088** 1.6 × 10^−3^	**0.243** 1.1 × 10^−18^	**0.468** 2.5 × 10^−70^	**0.185** 2.8 × 10^−11^	–		
Attention (6)^a^	5.89 ± 0.32	5.72 ± 0.56	5.49 ± 0.90	5.76 ± 0.59	5.39 ± 1.37	**0.104** 2.1 × 10^−4^	**0.171** 7.5 × 10^−10^	**0.395** 5.5 × 10^−49^	**0.323** 2.0 × 10^−32^	**0.227** 2.1 × 10^−16^	–	
Age	67.4 ± 11.0	63.9 ± 7.4	64.4 ± 10.2	67.8 ± 9.8	72.8 ± 6.1	−**0.190** 7.4 × 10^−12^	−**0.258** 6.4 × 10^−21^	−**0.160** 8.3 × 10^−9^	−**0.090** 1.3 × 10^−3^	−**0.088** 1.6 × 10^−3^	0.011 6.9 × 10^−1^	–

a Maximum score or subscore on assessment.

b Correlations calculated with missing-value cases excluded pairwise, bold indicates significance at the 0.01 level (two-tailed). UPSIT: University of Pennsylvania Smell Identification Test. HVLT: Hopkins Verbal Learning Test. MoCA: Montreal Cognitive Assessment Test. Visu-Exec: MoCA subscore for visuospatial and executive function. Delayed-Recall: MoCA subscore for delayed recall. PPMI-defined diagnostic groups: Asymptomatic-genetic-Parkinson's-disease subjects have a mutation, or are a first-degree relative of an individual having a mutation, in *LRRK2*, *SNCA*, or *GBA*; Symptomatic-genetic-Parkinson's-disease subjects have a mutation in *LRRK2*, *SNCA*, or *GBA*; Possible-prodromal-Parkinson's-disease subjects have REM-behavior sleep disorder and/or hyposmia.

Bonferroni post-hoc analyses evaluated significant differences in mean scores between pairs of diagnostic categories (Table 2). Healthy controls had higher means on all tests than subjects in symptomatic diagnostic categories, and higher means on all tests except the HVLT than asymptomatic-genetic-PD subjects. In turn, asymptomatic-genetic-PD subjects had means higher than those in all symptomatic diagnostic categories, except for having lower Delayed-Recall means than sporadic-PD subjects. Symptomatic-genetic-PD subjects, compared to sporadic-PD subjects, had lower UPSIT, MoCA and Attention means, higher VisuExec means, and similar HVLT and Delayed-Recall means. Possible-prodromal-PD subjects had means similar to or lower than those in all other diagnostic categories, except for having higher Visu-Exec means than sporadic-PD subjects. PPMI-inclusion criteria may contribute to differences between some diagnostic categories: inclusion criteria for healthy controls specified MoCA ≥26 and they had greater MoCA means than all other diagnostic categories; symptomatic-genetic-PD subjects with disease duration ≤7 years had lower UPSIT, MoCA, and Attention means than sporadic-PD subjects with disease duration ≤2 years; possible-prodromal-PD subjects had RBD and/or hyposmia.

**Table 2:** ANOVA Bonferroni post-hoc analysis.

	Healthy controls	Asymptomatic genetic Parkinson's disease	Symptomatic genetic Parkinson's disease	Sporadic Parkinson's disease	Possible prodromal Parkinson's disease
A. UPSIT					
Healthy controls	–				
Asymptomatic genetic Parkinson's disease	−1.06 ± 0.67 0.82 to −2.94 1.000	–			
Symptomatic genetic Parkinson's disease	−12.56* ± 0.72 −14.58 to −10.53 <0.001	−11.50* ± 0.65 −13.32 to −9.68 <0.001	–		
Sporadic Parkinson's disease	−10.56* ± 0.62 −12.36 to −8.89 <0.001	−9.57* ± 0.53 −11.07 to −8.07 <0.001	1.93* ± 0.60 0.26 to 3.61 0.012	–	
Prodromal	−16.84* ± 1.08 −19.85 to −13.81 <0.001	−15.78* ± 1.03 −18.67 to −12.89 <0.001	−4.28* ± 1.06 −7.27 to −1.29 0.001	−6.21* ± 1.00 −9.01 to −3.41 <0.001	–

B. HVLT					
Healthy controls	–				
Asymptomatic genetic Parkinson's disease	0.43 ± 0.46 −0.86 to 1.73 1.000	–			
Symptomatic genetic Parkinson's disease	−1.99* ± 0.50 −3.38 to −0.60 0.001	−2.43* ± 0.45 −3.68 to −1.17 <0.001	–		
Sporadic Parkinson's disease	−1.59* ± 0.43 −2.78 to −0.39 0.002	−2.02* ± 0.37 −3.05 to −0.99 <0.001	0.41 ± 0.41 −0.75 to 1.56 1.000	–	
Prodromal	−4.012* ± 0.74 −6.10 to −1.93 <0.001	−4.45* ± 0.71 −6.44 to −2.46 <0.001	−2.02 ± 0.73 −4.08 to 0.03 0.058	−2.43* ± 0.69 −4.36 to −0.50 0.004	–

C. MoCA.					
Healthy controls	–				
Asymptomatic genetic Parkinson's disease	−1.53* ± 0.23 −2.17 to −0.88 <0.001	–			
Symptomatic genetic Parkinson's disease	−2.28* ± 0.25 −2.97 to −1.59 <0.001	−0.75* ± 0.22 −1.38 to −0.13 0.007	–		
Sporadic Parkinson's disease	−1.10* ± 0.21 −1.69 to −0.50 <0.001	0.43 ± 0.18 −0.08 to 0.94 0.187	1.18* ± 0.20 0.61 to 1.76 <0.001	–	
Prodromal	−2.01* ± 0.37 −3.04 to −0.98 <0.001	−0.48 ± 0.35 −1.47 to 0.51 1.000	0.27 ± 0.36 −0.75 to 1.29 1.000	−0.91 ± 0.34 −1.87 to 0.05 0.076	–

D. MoCA subscore for visuospatial executive function					
Healthy controls	–				
Asymptomatic genetic Parkinson's disease	−0.30* ± 0.08 −0.52 to −0.08 0.001	–			
Symptomatic genetic Parkinson's disease	−0.43* ± 0.83 −0.67 to −0.20 <0.001	0.13 ± 0.08 −0.08 to 0.34 0.783	–		
Sporadic Parkinson's disease	−0.17 ± 0.07 −0.37 to 0.03 0.193	−0.13 ± 0.06 −0.31 to 0.04 0.301	−0.27* ± 0.07 −0.46 to −0.07 0.001	–	
Prodromal	−0.62* ± 0.13 −0.97 to −0.27 <0.001	0.32 ± 0.12 −0.01 to 0.66 0.070	0.19 ± 0.12 −0.16 to 0.54 1.000	0.46* ± 0.12 0.13 to 0.78 <0.001	–

E. MoCA subscore for delayed recall					
Healthy controls	–				
Asymptomatic genetic Parkinson's disease	−0.85* ± 0.13 −1.21 to −0.48 <0.001	–			
Symptomatic genetic Parkinson's disease	−0.66* ± 0.14 −1.05 to −0.27 <0.001	0.18 ± 0.13 −0.17 to 0.53 1.000	–		
Sporadic Parkinson's disease	−0.53* ± 0.12 −0.87 to −0.20 <0.001	0.31* ± 0.10 0.03 to 0.60 0.023	0.13 ± 0.12 −0.19 to 0.45 1.000	–	
Prodromal	−0.57 ± 0.21 −1.15 to 0.01 0.057	0.27 ± 0.20 −0.28 to 0.83 1.000	0.09 ± 0.20 −0.49 to 0.66 1.000	−0.04 ± 0.19 −0.58 to 0.50 1.000	–

F. MoCA subscore for attention					
Healthy controls	–				
Asymptomatic genetic Parkinson's disease	−0.18* ± 0.06 −0.35 to 0.00 0.044	–			
Symptomatic genetic Parkinson's disease	−0.40* ± 0.07 −0.59 to −0.22 <0.001	−0.23* ± 0.06 −0.40 to −0.06 0.001	–		
Sporadic Parkinson's disease	−0.13 ± 0.06 −0.29 to 0.02 0.178	0.04 ± 0.49 −0.10 to 0.18 1.000	0.27* ± 0.06 0.12 to 0.42 <0.001	–	
Prodromal	−0.50* ± 0.10 −0.78 to −0.22 <0.001	−0.33* ± 0.09 −0.59 to −0.06 0.006	−0.10 ± 0.10 −0.37 to 0.18 1.000	−0.37* ± 0.09 −0.62 to −0.11 <0.001	–

For the test indicated, the rows of each cell list the mean difference ± standard error (*P < .05), 95% confidence interval, and *P* value for the indicated pair of diagnostic categories. UPSIT: University of Pennsylvania Smell Identification Test. HVLT: Hopkins Verbal Learning Test. MoCA: Montreal Cognitive Assessment Test. PPMI-defined diagnostic groups: Asymptomatic genetic-Parkinson's-disease subjects have a mutation, or are a first-degree relative of an individual having a mutation, in *LRRK2*, *SNCA*, or *GBA*; Symptomatic-genetic-Parkinson's-disease subjects have a mutation in *LRRK2*, *SNCA*, or *GBA*; Possible-prodromal-Parkinson's-disease subjects have REM-behavior sleep disorder and/or hyposmia.

### 3.3 The indirect effects of cognitive processes

Before building SEM models to evaluate the indirect effects of cognitive processes, we confirmed that PPMI diagnostic categories are predictive of UPSIT scores by regressing UPSIT scores on the PPMI diagnostic categories. Mirroring the ANOVA results, UPSIT scores in the sporadic-PD, asymptomatic-genetic-PD, symptomatic-genetic-PD and possible-prodromal-PD diagnostic categories were significantly different from those in the healthy-control category. Specifically, healthy controls had UPSIT scores closest to asymptomatic-genetic-PD (*b* = −1.05, *Standard Error* (*SE*) = 0.44, *z* = −2.38, *P* = .017) while symptomatic individuals showed larger differences [symptomatic-genetic-PD (*b* = −12.56, *SE* = 0.69, *z* = −18.21, *P* < .001), sporadic-PD (*b* = −10.62, *SE* = 0.52, *z* = −20.44, *P* < .001) and possible-prodromal-PD (*b* = −16.84, *SE* = 0.90, *z* = −18.69, *P* < .001)]. Thus, subjects in all symptomatic diagnostic categories had poorer olfactory function than healthy controls. Overall, the five diagnostic categories explained 36.6% of the variance in UPSIT scores.

We also used regression analysis to verify that the potentially mediating variables (MoCA and HVLT) are predictive of UPSIT scores after controlling for associations between HVLT and MoCA scores. MoCA scores (*b* = 0.07, *SE* = 0.03, *z* = 2.31, *P* = .021) and HVLT scores (*b* = 0.25, *SE* = 0.03, *z* = 8.46, *P* < .001) significantly predicted UPSIT scores and together explained 8.3% of the variance in UPSIT scores. In separate, simple regressions of UPSIT on MoCA and UPSIT on HVLT, the MoCA was a significant predictor of the UPSIT (*b* = 0.18, *SE* = 0.03, *z* = 6.54, *P* < .001), as was the HVLT (*b* = 0.28, *SE* = 0.03, *z* = 10.88, *P* < .001). Though each individually was a significant predictor of the UPSIT, when both were considered as joint predictors, the MoCA lost more of its predictive magnitude and strength than did the HVLT.

**Fig. 2:**
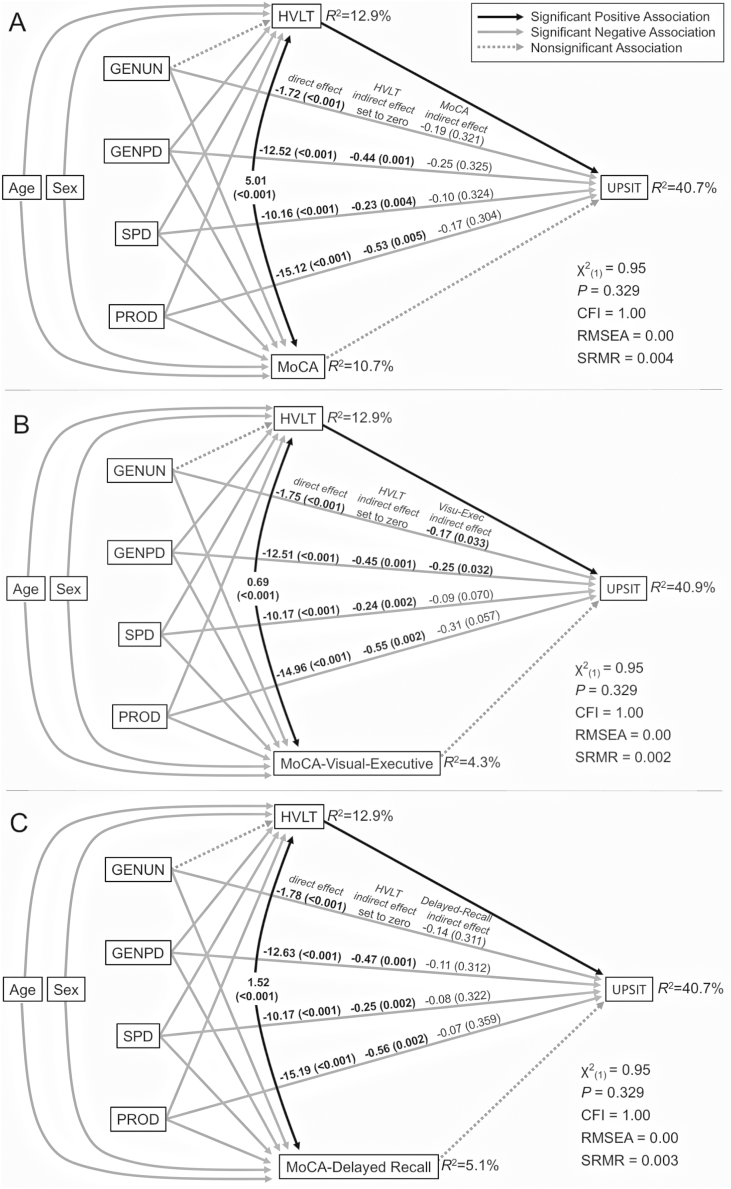
Structural equation models assessing the indirect effects of cognitive measures. Models reveal the magnitude and strength of the indirect effect of cognitive measures on the relationship between UPSIT and the diagnostic categories, accounting for age and sex as covariates. Since the indirect effects between GENUN and HVLT in each model were non-significant, the associations between GENUN and HVLT were fixed to zero to give the models one degree of freedom. Significant and nonsignificant associations between study variables are depicted by connecting lines as described in the legend. The three sets of values above the lines between diagnostic categories and UPSIT convey the magnitude (and strength, as *p*-value), in order, of their direct association, the indirect association of the HVLT along the arrowed lines from the diagnostic category to HVLT to UPSIT, and, the indirect association of the second cognitive measure along the arrowed lines from the diagnostic category to that measure to UPSIT. Fit statistics and the variance (*R*^2^) in scores explained by the models are indicated. (A) Model assessing the indirect effects of the HVLT and MoCA. (B) Model assessing the indirect effects of the HVLT and the visuospatial-executive-functioning subscore of the MoCA. (C) Model assessing the indirect effects of the HVLT and the delayed-memory-recall subscore of the MoCA. Model statistics: χ^2^ = model chi-square, CFI = comparative fit index, RMSEA = root mean square error of approximation, SRMR = standard root mean square residual. PPMI-defined diagnostic categories: HC = healthy controls; GENUN = individuals with asymptomatic genetic (*LRRK2*, *SNCA*, or *GBA*) PD; GENPD = individuals with symptomatic genetic PD; SPD = individuals with sporadic PD at baseline; PROD (possible prodromal PD) = individuals diagnosed with hyposmia and/or RBD.

Since the diagnostic categories, HVLT scores, and MoCA scores were all predictive of UPSIT scores, we built an SEM model to assess whether HVLT and MoCA scores have indirect effects on the relationship between the diagnostic categories and UPSIT scores. This model compared each of the PD-related diagnostic categories to healthy controls, included UPSIT scores regressed onto all diagnostic categories, the indirect effects of HVLT and MoCA scores on these relationships, and the effects of the age and sex as covariates, since olfactory function declines during healthy aging and differs between sexes. The relationship between HVLT scores and the asymptomatic-genetic-PD diagnostic category was not significant, so this association was set to zero to give the model one degree of freedom. That had no effect on other model relationships. The resulting model (Fig. 2A) was a good fit to the data (χ^2^_(1)_ = 0.95, *P* = .329, CFI = 1.00, RMSEA = 0.00, SRMR = 0.004) and explained 40.7% of the variability in UPSIT, 12.9% in HVLT and 10.7% in MoCA scores. Thus, 40.7% of the variability in UPSIT scores is attributable to age, sex, diagnostic category, HVLT scores and MoCA scores.

In this model, MoCA scores do not have significant indirect effects on the relationships between each of the four non-healthy-control diagnostic categories and UPSIT scores. In contrast, HVLT scores have significant indirect effects on the relationships between all symptomatic diagnostic categories and UPSIT scores. Importantly, this model indicates that HVLT scores provide a better explanation for UPSIT scores in symptomatic diagnostic categories than do MoCA scores.

Given our and others' [27,28] results documenting the relationship between MoCA scores and olfactory function, we assessed the magnitude of the indirect effects of MoCA scores without HVLT scores. UPSIT scores were regressed against the asymptomatic-genetic-PD, symptomatic-genetic-PD, sporadic-PD and possible-prodromal-PD diagnostic categories, comparing them to healthy controls with age and sex as covariates, and using MoCA scores as a mediating variable. The resulting regression model was significant (*F*_7,1268_ = 120.6, *P* = 1 × 10^−135^, *R*^2^ = 0.4) and explained 40% of the variability in UPSIT scores and 11% of the variability in MoCA scores. Age, sex, the non-healthy-control diagnostic categories, and MoCA scores were significant predictors of UPSIT scores (Supplemental Table 2). MoCA scores have significant indirect effects on the relationship between UPSIT scores and the diagnostic categories of asymptomatic-genetic-PD (*b* = −0.45, *P* = .010), symptomatic-genetic-PD (*b* = −0.63, *P* = .012), sporadic-PD (*b* = −0.27, *P* = .012), and possible-prodromal-PD (*b* = −0.42, *P* = .013). Thus, MoCA scores do have significant indirect effects, but they are no longer significant when HVLT scores also are included in the model. Therefore, the HVLT more effectively captures the indirect effects of cognition on the relationship between a diagnostic category and UPSIT than does the MoCA.

To determine whether MoCA subscores for Visu-Exec, Attention or Delayed-Recall, by themselves, have significant indirect effects, we replaced total MoCA scores with these subscores and tested them using regression analyses. Only the Visu-Exec and Delayed-Recall subscores were significant predictors of UPSIT scores. We then built SEM models to evaluate the indirect effects of (1) Visu-Exec, (2) Delayed-Recall (3) Visu-Exec and HVLT and (4) Delayed-Recall and HVLT.

Visu-Exec had significant indirect effects on the relationship between UPSIT and the diagnostic categories of asymptomatic-genetic-PD (*b* = −0.22, *P* = .008), symptomatic-genetic-PD (*b* = −0.32, *P* = .007), sporadic-PD (*b* = −0.12, *P* = .030), and possible-prodromal-PD (*b* = −0.45, *P* = .017). An SEM model with Visu-Exec and HVLT scores had a good fit to the data (χ^2^_(1)_ = 0.95, *P* = .329, CFI = 1.00, RMSEA = 0.00, SRMR = 0.002) and explained 40.9% of the variability in UPSIT scores, 12.9% of the variability in HVLT scores and 4.3% of the variability in Visu-Exec subscores (Fig. 2B). It revealed 1) significant indirect associations of the HVLT on the relationships between the UPSIT and the three symptomatic diagnostic categories and 2) significant indirect effects of the Visu-Exec on the relationships between the UPSIT and the genetic diagnostic categories, with the other symptomatic diagnostic categories approaching significance. As found for the SEM model including the HVLT and MoCA, the HVLT also had stronger indirect effects on the symptomatic-genetic-PD diagnostic category than the Visu-Exec. Strikingly, Visu-Exec subscores provide a better explanation for UPSIT scores in the genetic diagnostic categories than do total MoCA scores.

Delayed-Recall subscores have significant indirect effects on the relationship between UPSIT scores and the diagnostic categories of asymptomatic-genetic-PD (*b* = −0.53, *P* = .017), symptomatic-genetic-PD (*b* = −0.35, *P* = .019), and sporadic-PD (*b* = −0.24, *P* = .026), but not possible-prodromal-PD (*b* = −0.01, *P* = .995). When incorporated into a SEM model that also includes HVLT scores, however, the indirect effects of Delayed-Recall subscores lose significance, just as we found for total MoCA scores when they were included in a model with HVLT scores (Fig. 2C).

## 4 Discussion

In PD, olfactory dysfunction is a common early manifestation [[2], [3], [4], [5], [6]] and cognitive dysfunction, specifically visuospatial and executive dysfunction, is also common [29,30]. Analysis of PPMI data shows that scores on tests of verbal learning/memory, global cognition, visuospatial-executive function, and delayed-recall are significant predictors of olfactory-function-test scores. Furthermore, SEM analysis demonstrates that in the symptomatic PPMI diagnostic categories, verbal learning/memory is a better indirect predictor of olfactory deficits than is global cognition. Furthermore, visuospatial and executive function also has significant indirect effects. We infer that the HVLT and Visu-Exec each assess cognitive processes that are also utilized by the UPSIT, and that impairment in those processes contribute to lowered olfactory-function-test scores in PD.

Both the UPSIT and HVLT are structured to utilize memory and/or memory retrieval. The UPSIT requires working and/or short-term memory processes to retain an odor memory while connecting it to semantic and/or episodic long-term memory to establish an association with its name. The HVLT presents auditory cues that must be retained in short-term memory so they can be repeated back. Efficient recall can be aided by making connections among the words using long-term memory. Scores on both tests will be influenced by working and/or short-term memory capacity/processes or memory retrieval. The significant indirect effects of HVLT scores for UPSIT scores on the symptomatic diagnostic categories may arise because the HVLT is a good measure for the use of these cognitive domains within the UPSIT.

Decision-making is a cognitive process utilized by the UPSIT, HVLT and Visu-Exec. A participant chooses between odor-choices in the UPSIT, strategies for remembering words (*e.g.*, mnemonic, chunking, or relatedness) and which words to say aloud in the HVLT, and how to draw lines in a specific direction, order, and location in the Visu-Exec. Their shared significant indirect effects for UPSIT scores on the symptomatic-genetic-PD diagnostic category likely result from impairment in potentially overlapping cognitive domains. We hypothesize that the indirect effects of the HVLT and Visu-Exec arise because these tests are good measures for decision-making processes also used within the UPSIT.

For the sporadic-PD and possible-prodromal-PD diagnostic categories, the HVLT has significant indirect effects, while the Visu-Exec indirect effects only approach significance. For the symptomatic-genetic-PD diagnostic category, both the HVLT and Visu-Exec each have significant indirect effects on the UPSIT. Differences in significance of the Visu-Exec indirect effects for different symptomatic diagnostic categories could reflect disease duration, severity, subtype, and/or genetic status: individuals in the symptomatic-genetic-PD diagnostic category are H&Y I-III while those in the sporadic-PD diagnostic category are H&Y I-II; those in the possible-prodromal-PD diagnostic category lack motor symptoms and all may not develop PD. Alternatively, the significant indirect effects of HVLT and Visu-Exec only for the symptomatic-genetic-PD diagnostic category may reflect these tests measuring a convergent cognitive process affecting the UPSIT only in that diagnostic category, and/or reflect a difference in the underlying neurodegenerative mechanism in genetic forms of PD.

Focused tests of memory and decision-making will be useful to better understand cognitive mechanisms contributing to perceived olfactory dysfunction. While the HVLT and Visu-Exec were strongly significant here, their effect sizes were relatively small. This may reflect the multiple levels at which olfactory dysfunction can occur. Longitudinal assessment to characterize the time course of impairment in specific cognitive domains relative to olfactory-function-test scores, and whether the strength of an indirect effect increases with disease severity and duration will provide insight into the etiology of both olfactory dysfunction and the neurodegenerative process.

The following are the supplementary data related to this article.

Supplemental Fig. 1 Differences in UPSIT, HVLT, and MoCA scores among diagnostic categories. Supplemental Fig. 1

Supplemental Fig. 2 Pairwise graphical comparison of univariate density estimates for study variables. Supplemental Fig. 2

Supplemental Table 1 Correlations between study variables for each diagnostic category compared to healthy controls. Supplemental Table 1

Supplemental Table 2 Regression model of UPSIT scores on diagnostic categories, sex, age and MoCA scores. Supplemental Table 2

## Financial disclosure

No disclosures or conflicts of interest related to the research in this manuscript.

## Funding sources for study

The authors of this study did not receive any specific grant from funding agencies in the public, commercial, or not-for-profit sectors.

## Declaration of Competing Interest

None.
