# Supplementary material for: Cognitive processes that indirectly affect olfactory dysfunction in Parkinson's disease
Source: Clin Park Relat Disord. 2019 Jul 20;1:13–20. doi: 10.1016/j.prdoa.2019.07.003 (PMC8288748; doi:10.1016/j.prdoa.2019.07.003)
Supplement: Supplemental Fig. 1 — Differences in UPSIT, HVLT, and MoCA scores among diagnostic categories. [file mmc1.pdf]

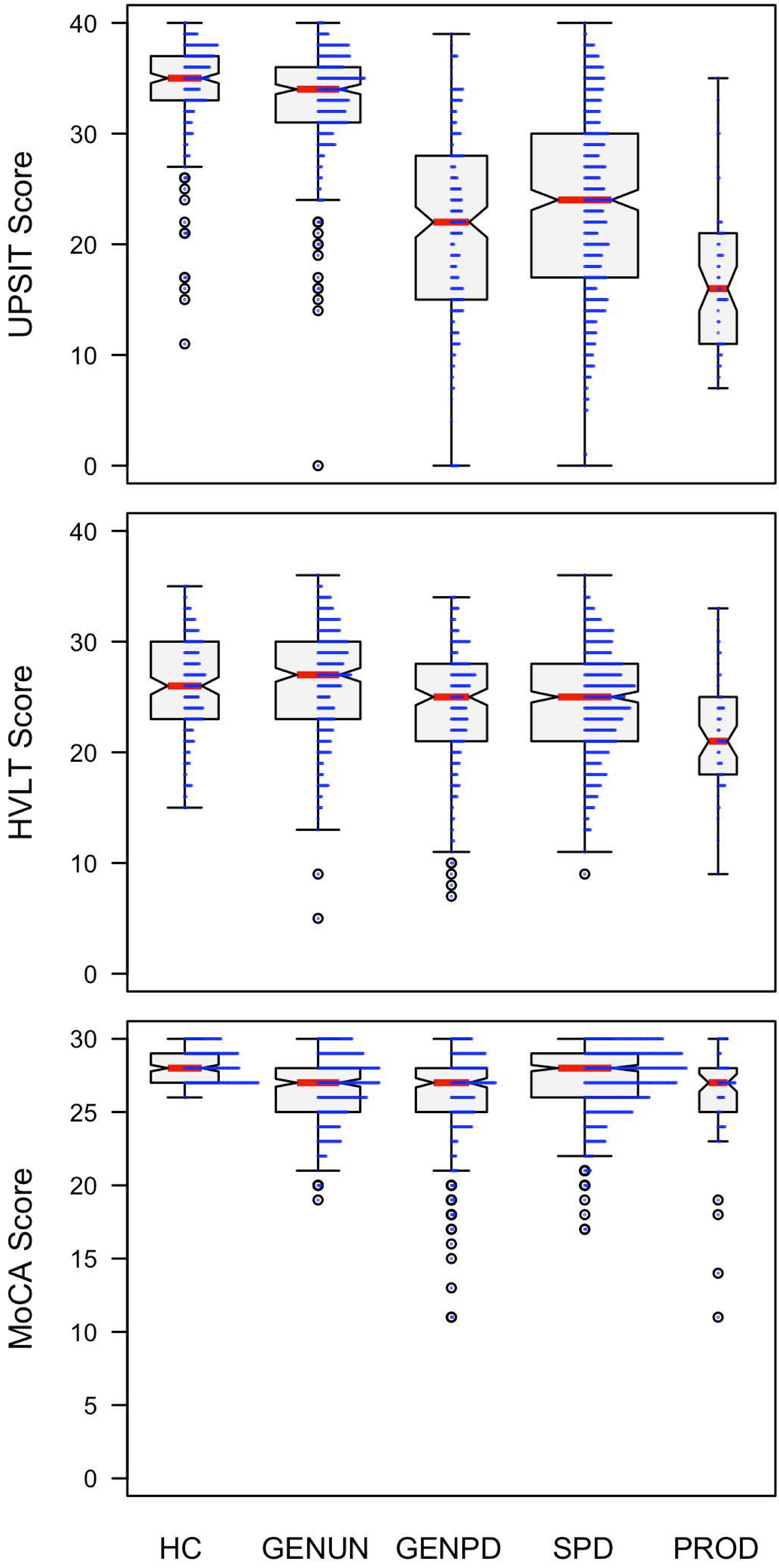

UPSIT Score
HVLT Score
MoCA Score
0
10
20
30
40
0
10
20
30
40
0
5
10
15
20
25
30
HC
GENUN
GENPD
SPD
PROD

Supplemental Figure 1. Differences in UPSIT, HVLT, and MoCA scores among diagnostic categories.

Each box shows the interquartile range, with box widths proportional to the number of subjects in each category. The notch indicates the 95% confidence interval around the median shown as a red line, the upper whisker is the minimum of either the maximum value or the 75th percentile plus 1.5 times the interquartile range, the lower whisker is the maximum of either the minimum value or the 25th percentile minus 1.5 times the interquartile range, the outliers are circled, and stacked blue points indicate the distribution of scores in each diagnostic category. PPMI-defined diagnostic categories: HC = healthy controls; GENUN = asymptomatic individuals having a mutation, or a first-degree relative of an individual having a mutation, in *LRRK2*, *SNCA*, or *GBA*; GENPD = symptomatic individuals having a mutation in *LRRK2*, *SNCA*, or *GBA*; SPD = individuals with sporadic PD at baseline; PROD = individuals with prodromal symptoms of PD.
