# Supplementary material for: Cognitive processes that indirectly affect olfactory dysfunction in Parkinson's disease
Source: Clin Park Relat Disord. 2019 Jul 20;1:13–20. doi: 10.1016/j.prdoa.2019.07.003 (PMC8288748; doi:10.1016/j.prdoa.2019.07.003)
Supplement: Supplemental Fig. 2 — Pairwise graphical comparison of univariate density estimates for study variables. [file mmc2.pdf]

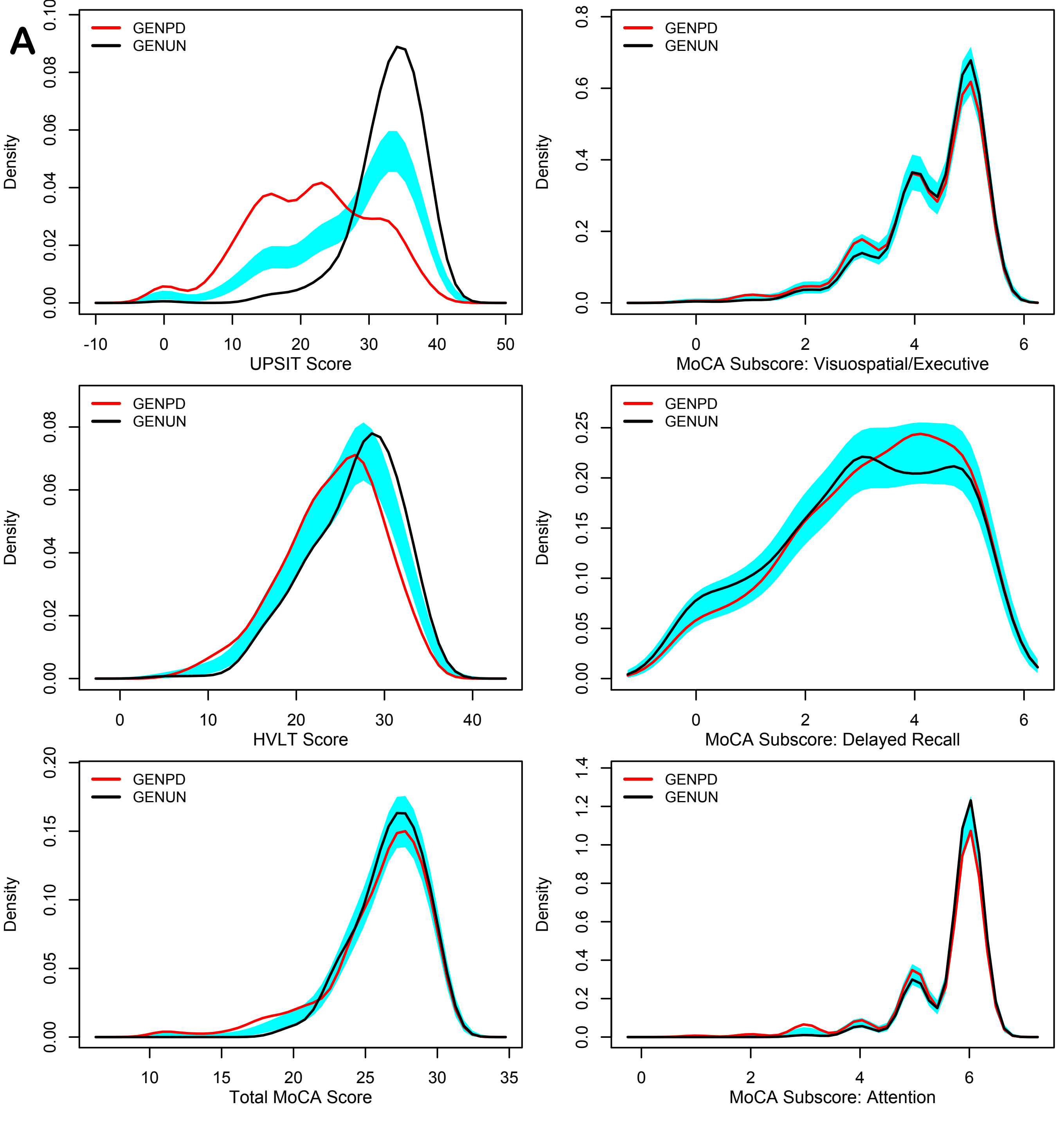
A
GENPD
GENUN
Density
UPSIT Score
MoCA Subscore: Visuospatial/Executive
HVLT Score
MoCA Subscore: Delayed Recall
Total MoCA Score
MoCA Subscore: Attention

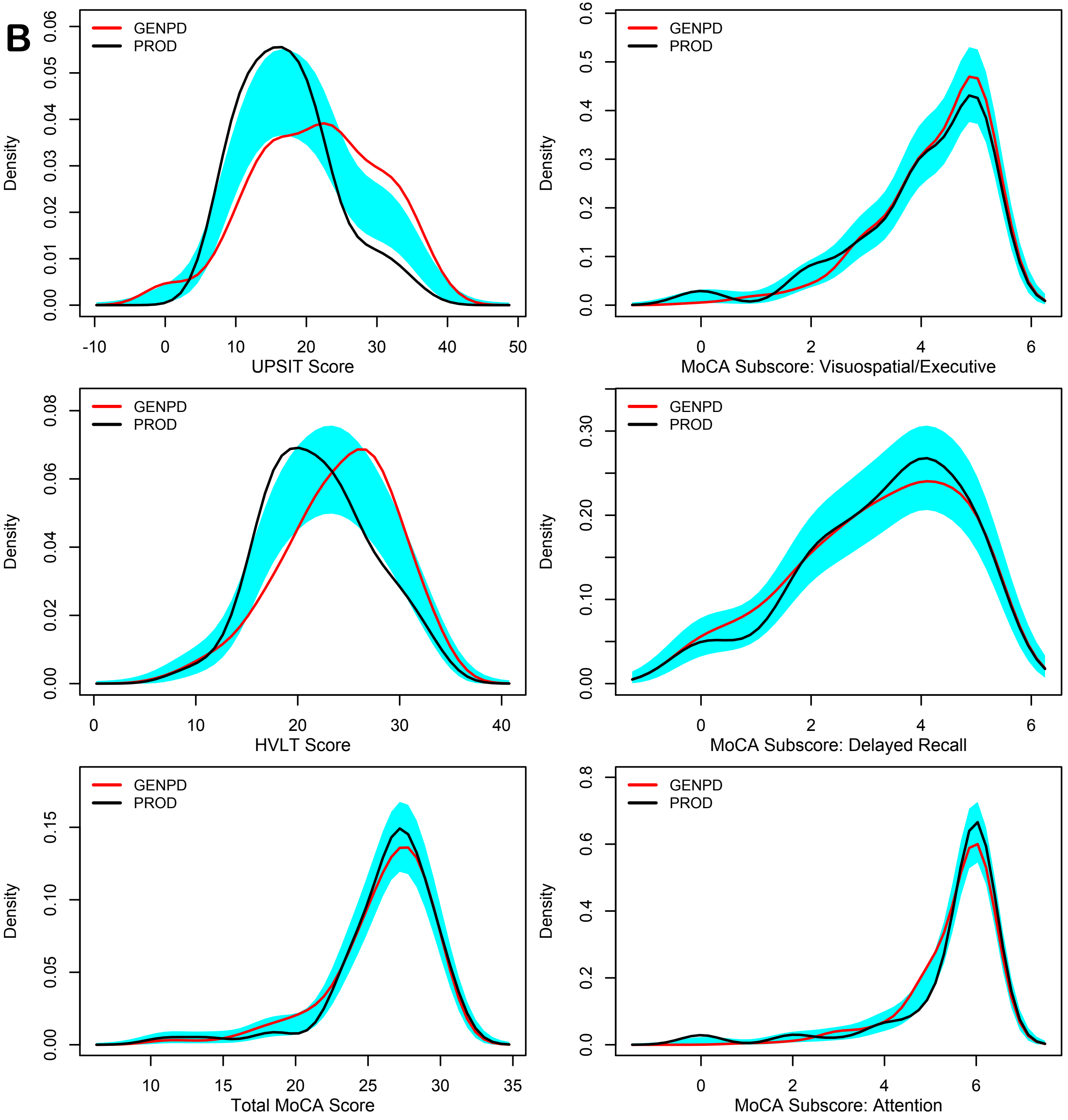

B
GENPD
PROD
Density
UPSIT Score
MoCA Subscore: Visuospatial/Executive
HVLT Score
MoCA Subscore: Delayed Recall
Total MoCA Score
MoCA Subscore: Attention

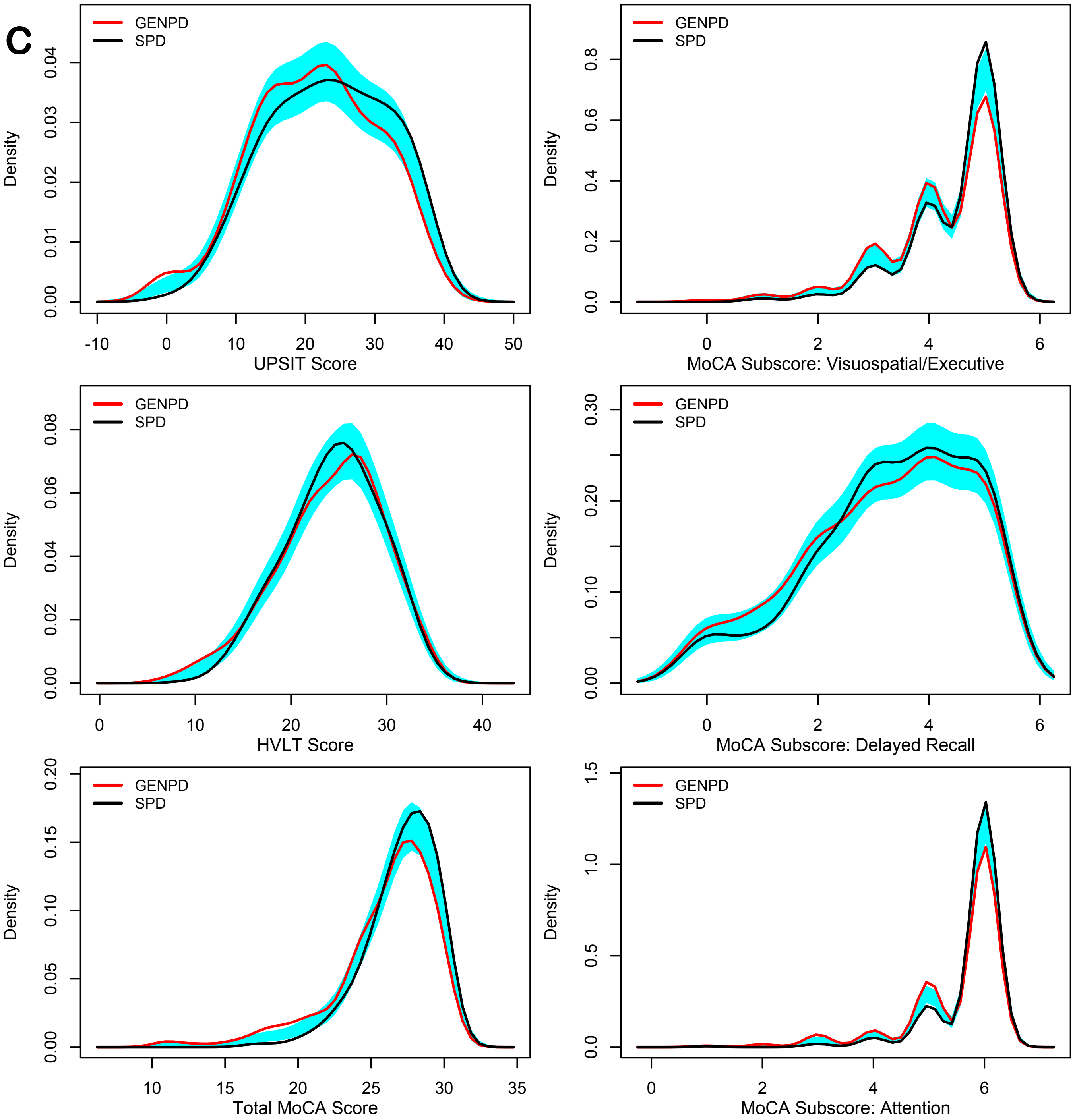
C
GENPD
SPD
Density
UPSIT Score
MoCA Subscore: Visuospatial/Executive
HVLT Score
MoCA Subscore: Delayed Recall
Total MoCA Score
MoCA Subscore: Attention

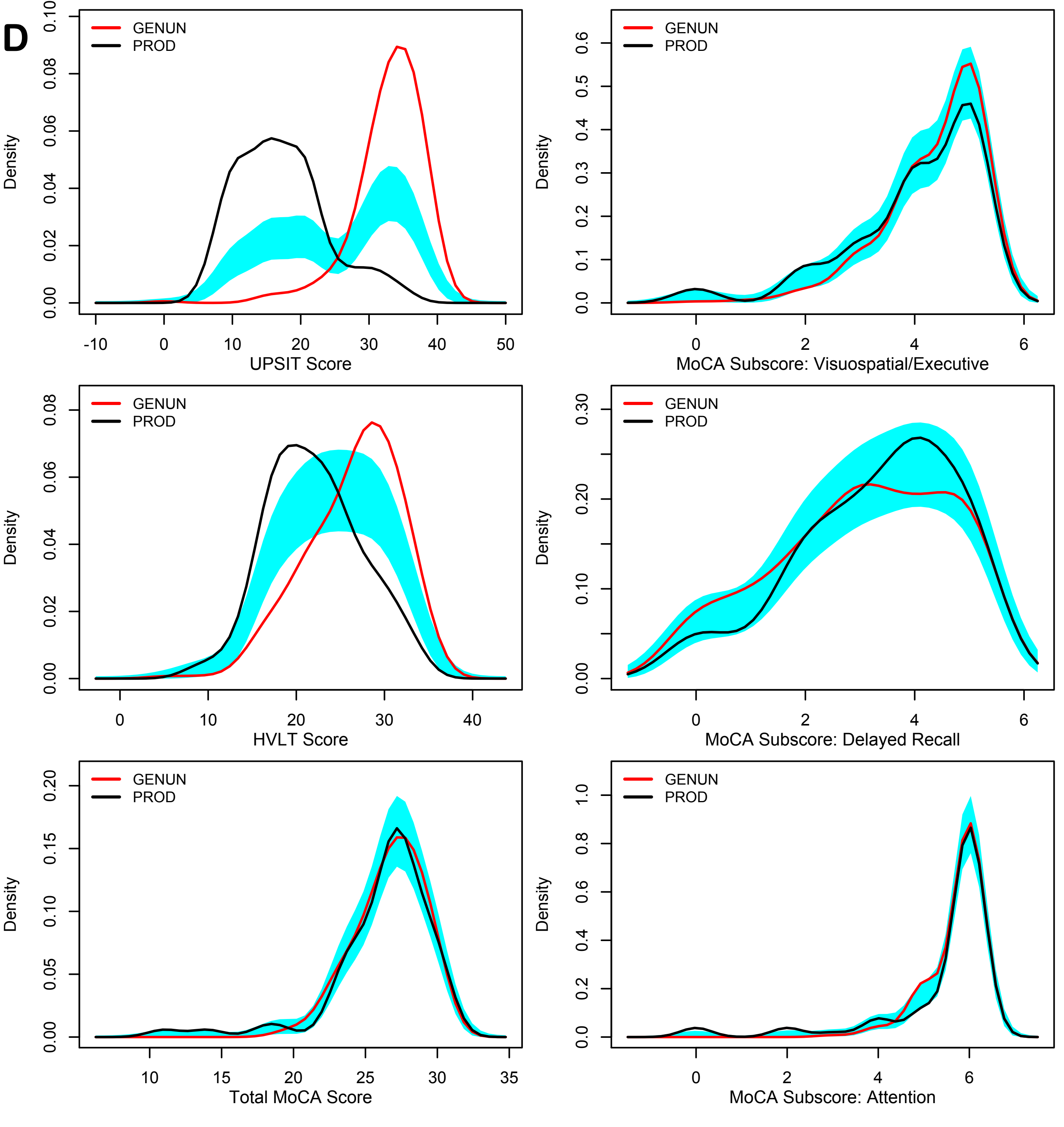
D
GENUN
PROD
Density
UPSIT Score
MoCA Subscore: Visuospatial/Executive
HVLT Score
MoCA Subscore: Delayed Recall
Total MoCA Score
MoCA Subscore: Attention

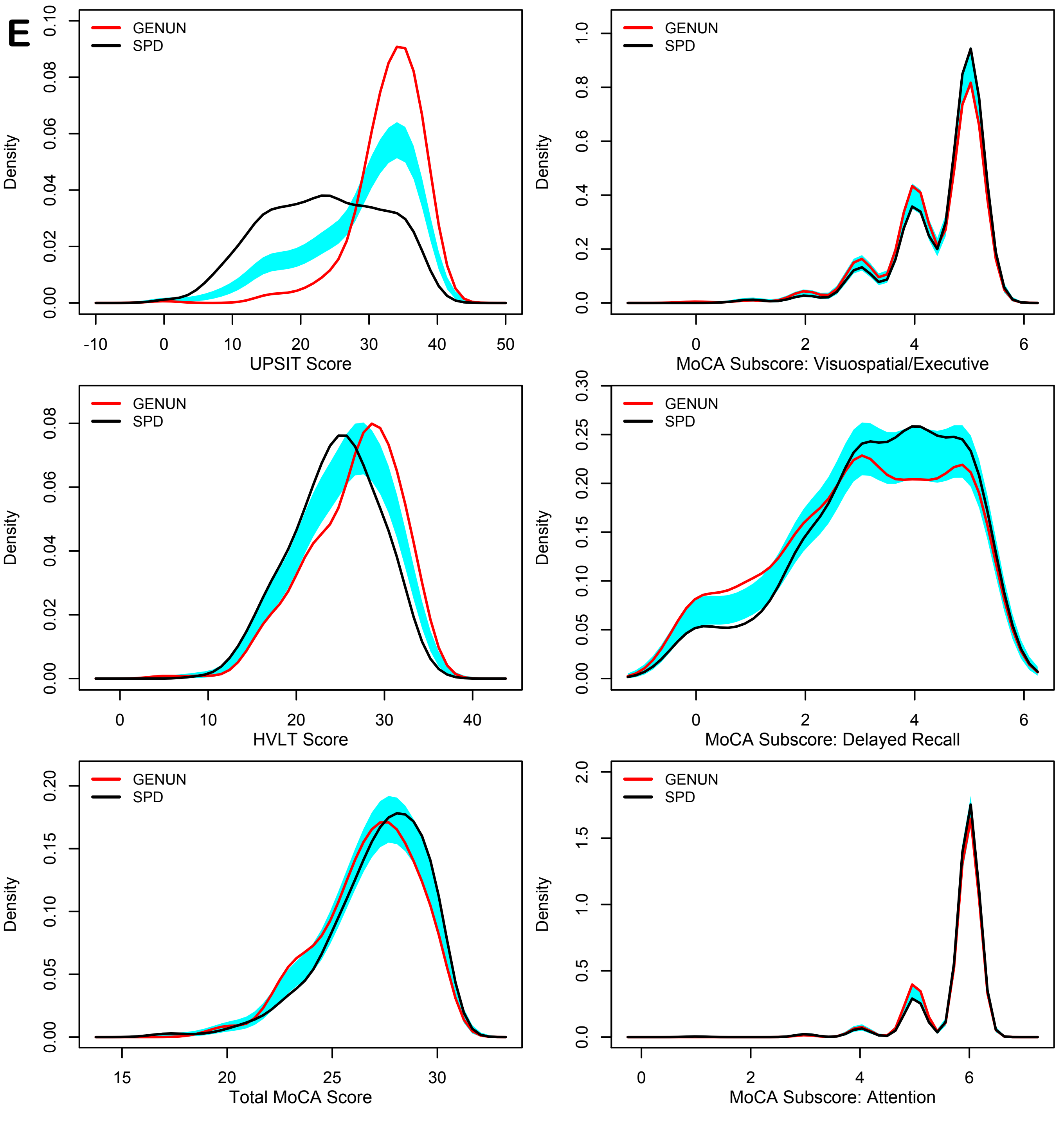
E
GENUN
SPD
Density
UPSIT Score
MoCA Subscore: Visuospatial/Executive
HVLT Score
MoCA Subscore: Delayed Recall
Total MoCA Score
MoCA Subscore: Attention

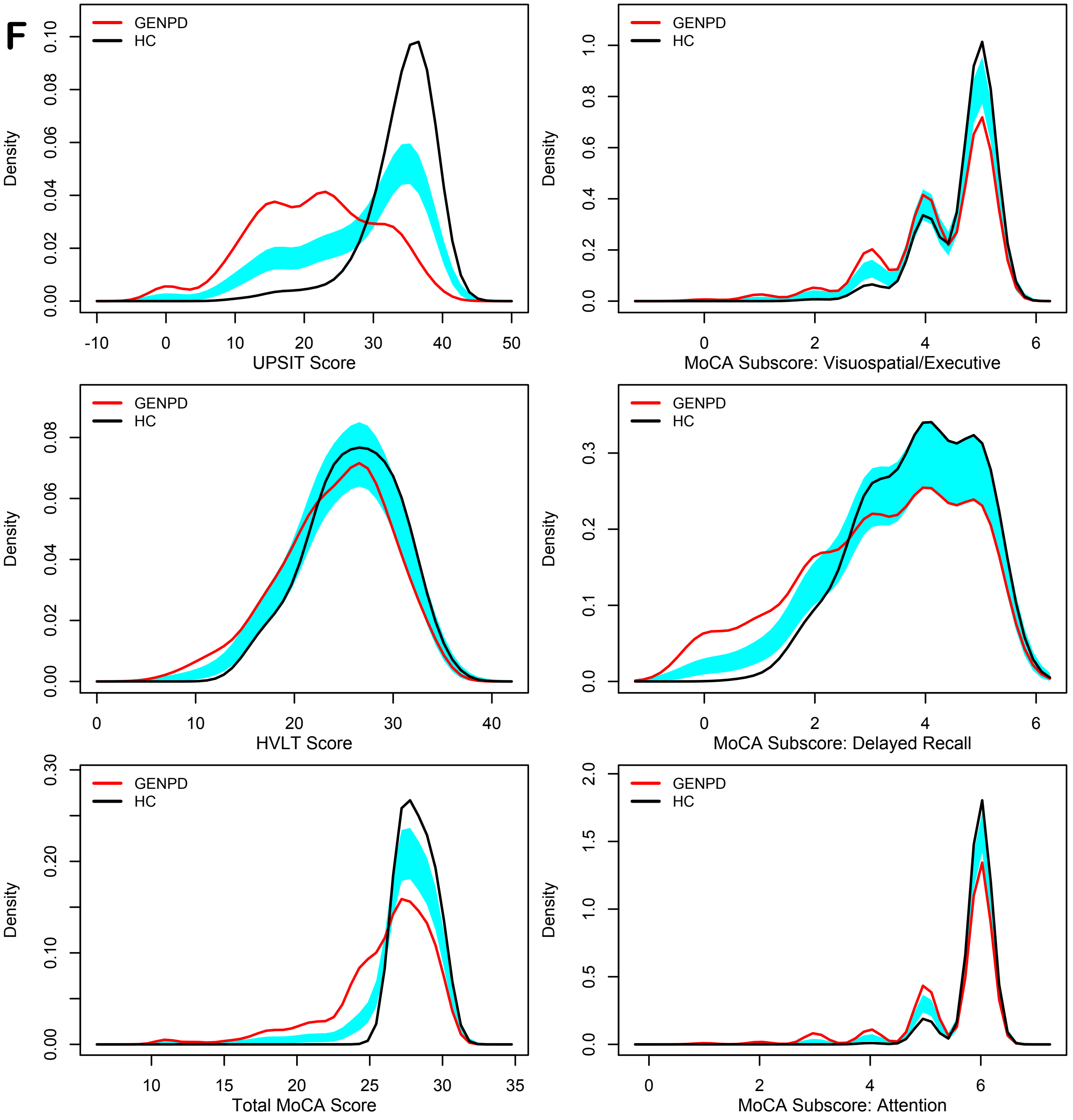

F
GENPD
HC
Density
UPSIT Score
MoCA Subscore: Visuospatial/Executive
HVLT Score
MoCA Subscore: Delayed Recall
Total MoCA Score
MoCA Subscore: Attention

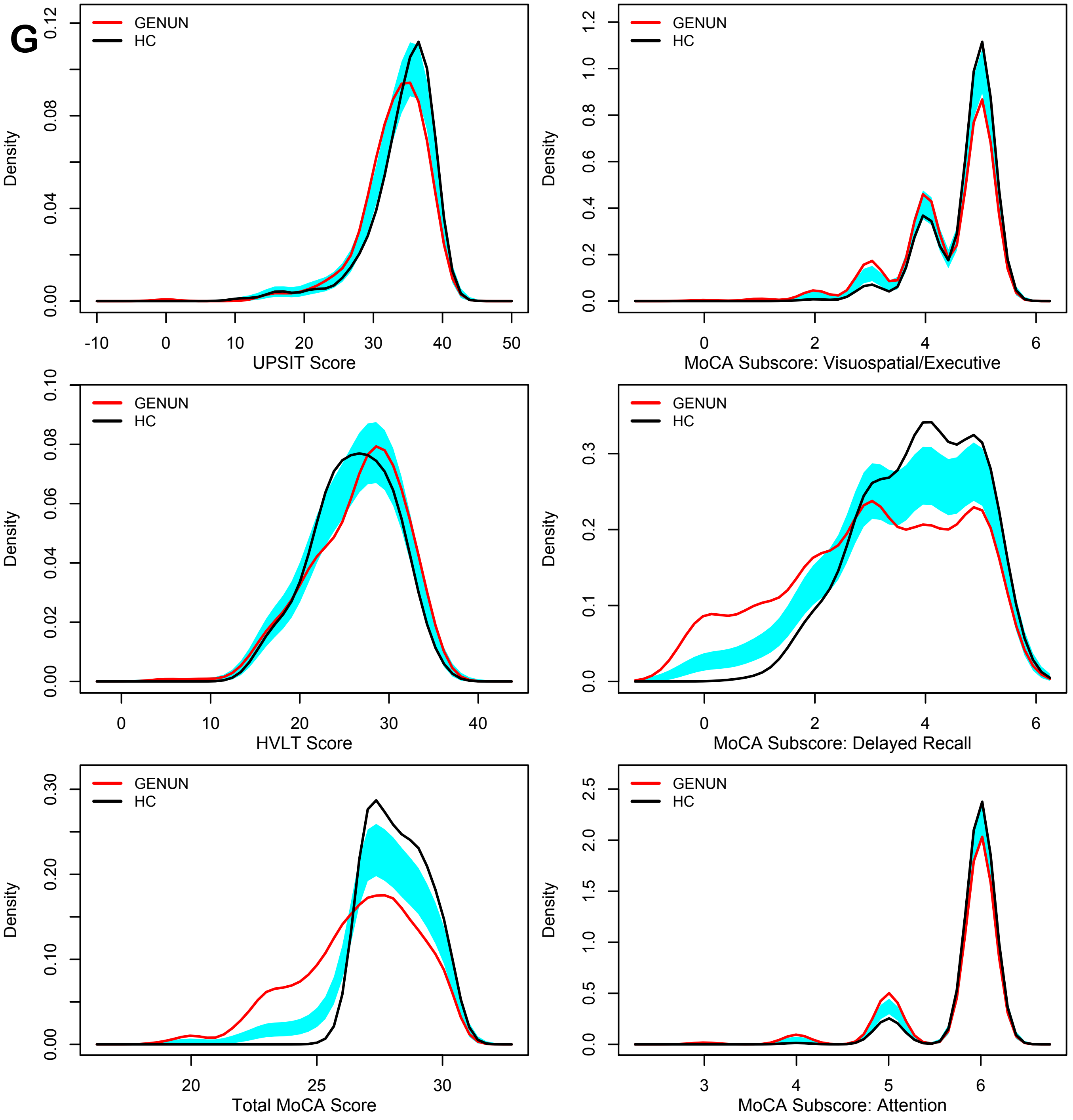

G
GENUN
HC
Density
UPSIT Score
MoCA Subscore: Visuospatial/Executive
HVLT Score
MoCA Subscore: Delayed Recall
Total MoCA Score
MoCA Subscore: Attention

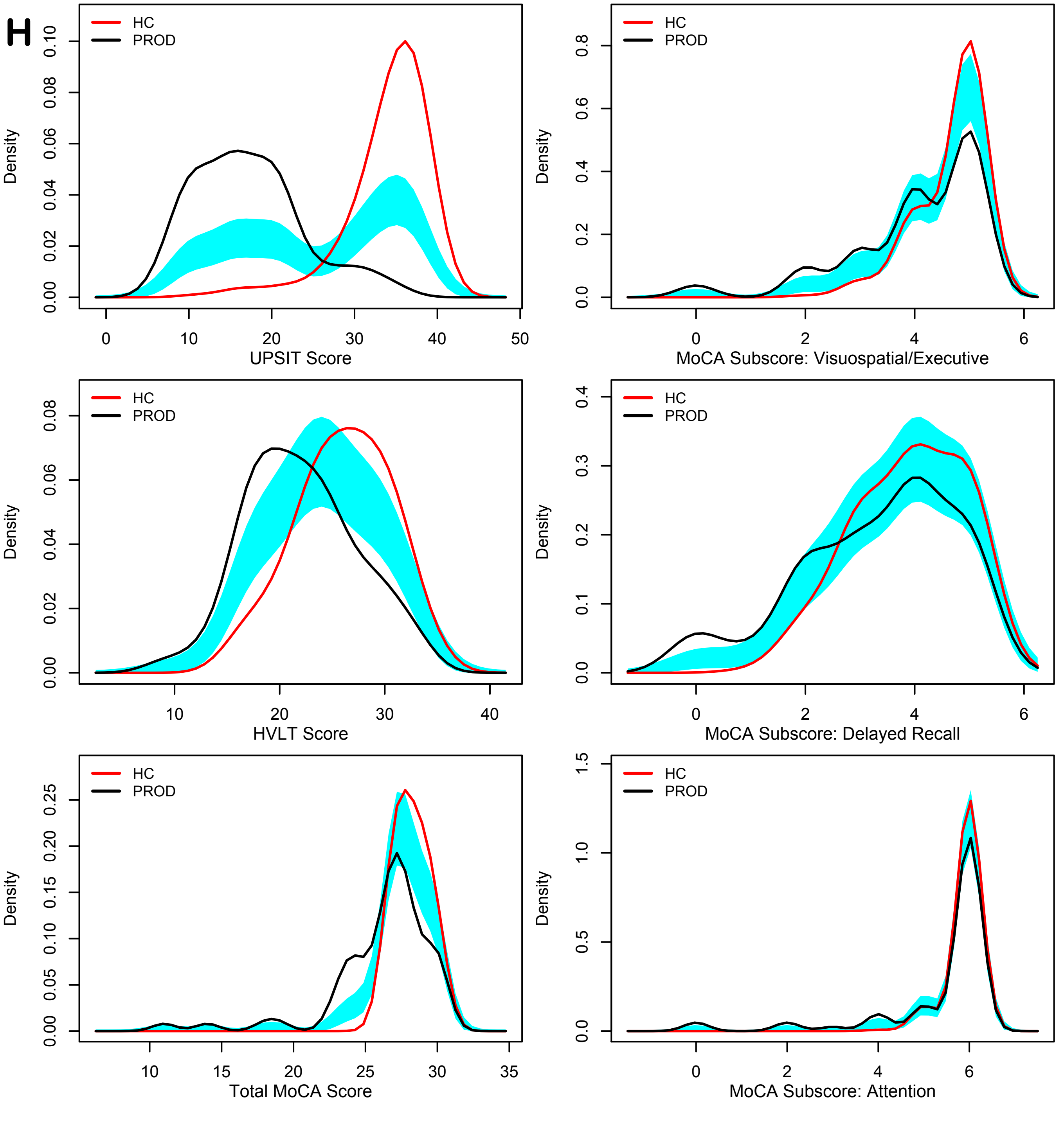

H
HC
PROD
Density
UPSIT Score
MoCA Subscore: Visuospatial/Executive
HVLT Score
MoCA Subscore: Delayed Recall
Total MoCA Score
MoCA Subscore: Attention

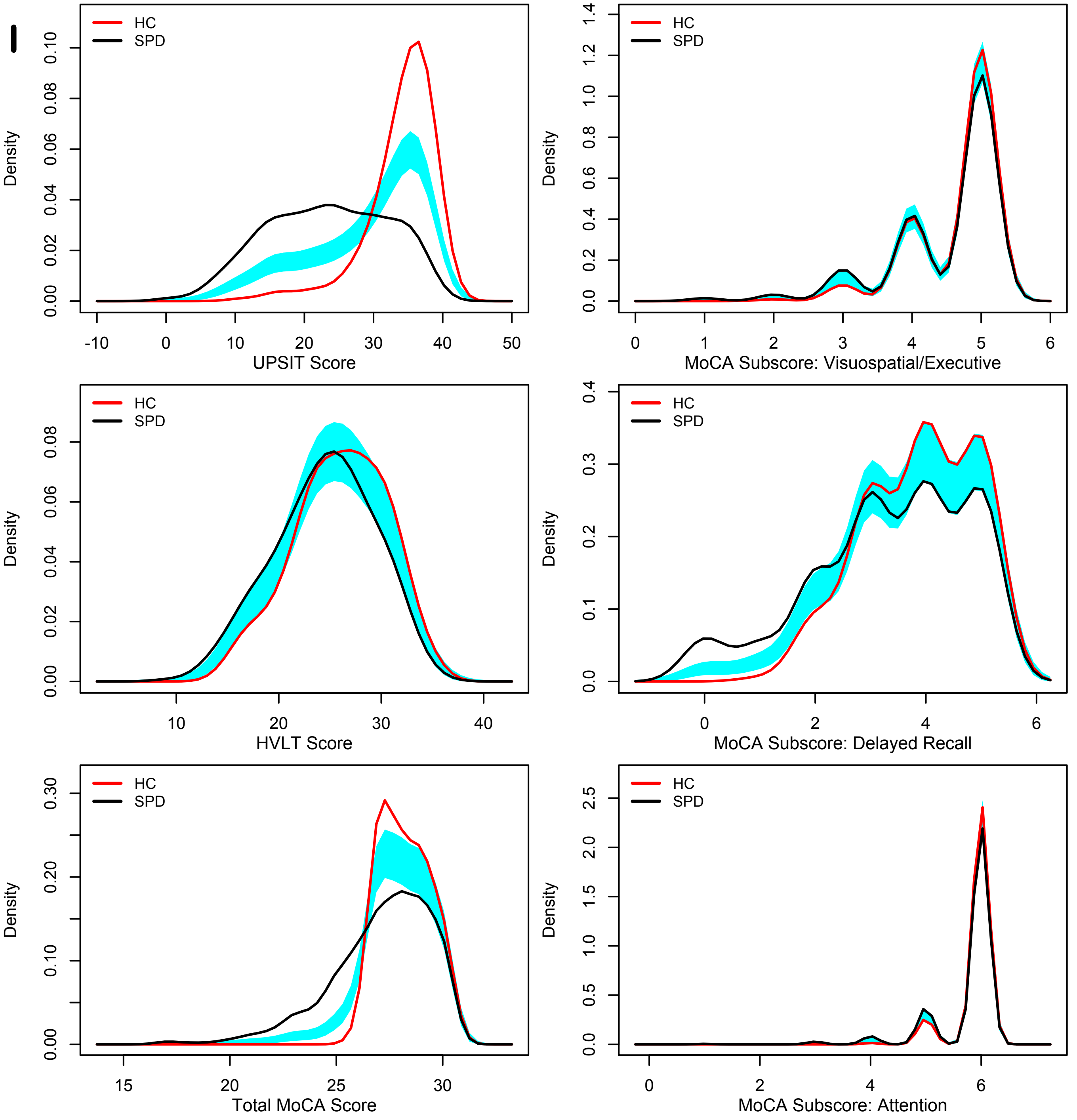
HC
SPD
Density
UPSIT Score
MoCA Subscore: Visuospatial/Executive
HVLT Score
MoCA Subscore: Delayed Recall
Total MoCA Score
MoCA Subscore: Attention

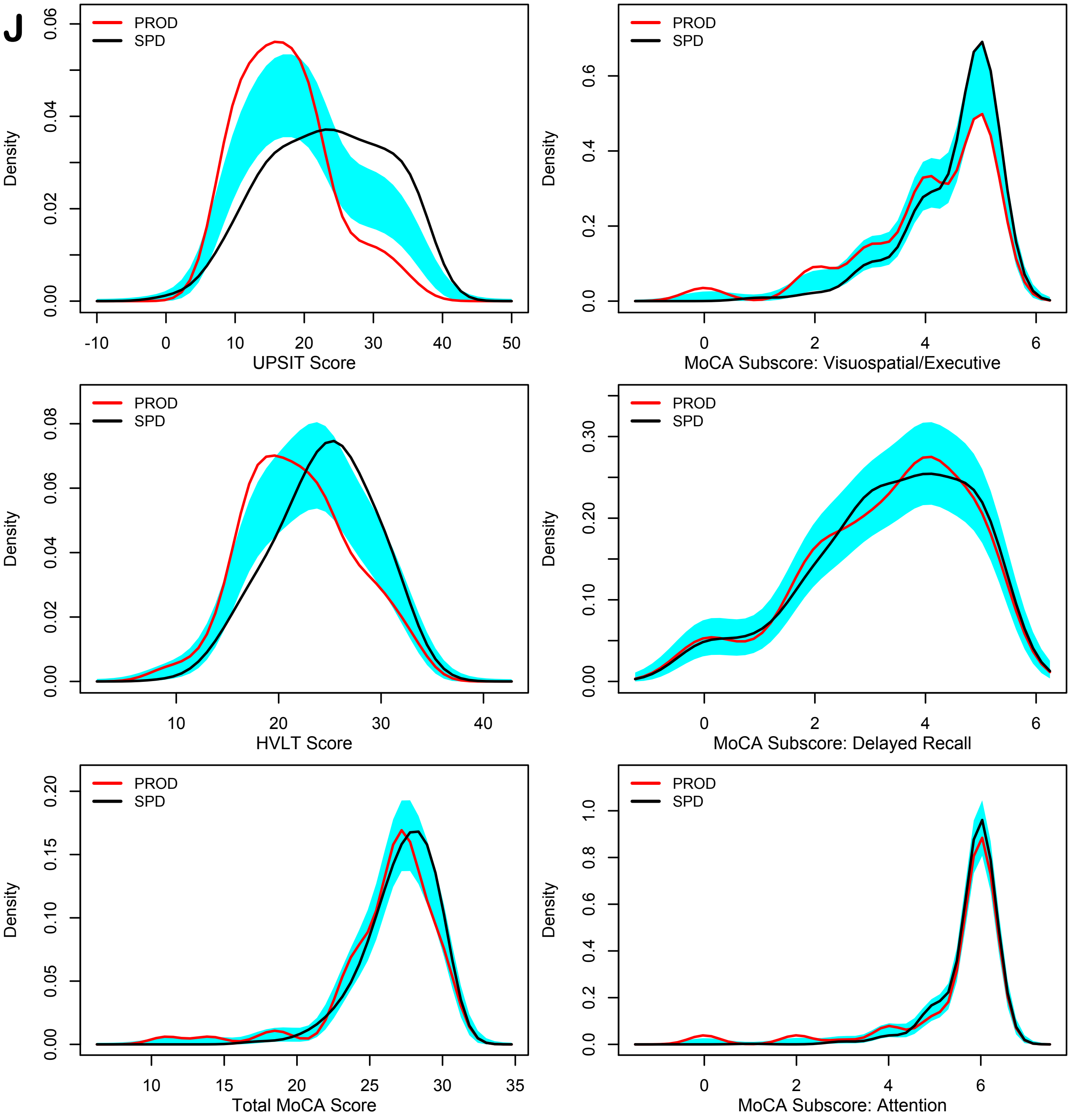

J
PROD
SPD
Density
UPSIT Score
MoCA Subscore: Visuospatial/Executive
HVLT Score
MoCA Subscore: Delayed Recall
Total MoCA Score
MoCA Subscore: Attention

Supplemental Figure 2. Pairwise graphical comparison of univariate density estimates for study variables.

(A) GENPD vs. GENUN. **(B)** GENPD vs. PROD. **(C)** GENPD vs. SPD. **(D)** GENUN vs. PROD. **(E)** GENUN vs. SPD. **(F)** HC vs. GENPD. **(G)** HC vs. GENUN. **(H)** HC vs. PROD. **(I)** HC vs. SPD. **(J)** SPD vs. PROD. Each part compares the density distributions of UPSIT scores, HVLT scores, MoCA scores, and MoCA subscores for Visu-Exec, Delayed-Recall and Attention for the indicated pair of diagnostic categories. The cyan region in each graph identifies a reference band, based on the standard error of the estimate, indicating where the density estimate is likely to lie if the data are normally distributed.

PPMI-defined diagnostic groups: HC = healthy controls; GENUN = asymptomatic individuals having a mutation, or a first-degree relative of an individual having a mutation, in *LRRK2*, *SNCA*, or *GBA*; GENPD = symptomatic individuals having a mutation in *LRRK2*, *SNCA*, or *GBA*; SPD = individuals with sporadic PD at baseline; PROD = Possible-prodromal-PD subjects that have REM-behavior sleep disorder and/or hyposmia.
