# Supplementary material for: Cognitive processes that indirectly affect olfactory dysfunction in Parkinson's disease
Source: Clin Park Relat Disord. 2019 Jul 20;1:13–20. doi: 10.1016/j.prdoa.2019.07.003 (PMC8288748; doi:10.1016/j.prdoa.2019.07.003)
Supplement: Supplemental Table 1 — Correlations between study variables for each diagnostic category compared to healthy controls. [file mmc3.docx]

Supplemental Table 1. Correlations between study variables for each diagnostic category compared to healthy controls.

|  | UPSIT | HVLT | Age | Diagnosis | Sex | MoCA | Visu-Exec | Delayed-  Recall |  |
| --- | --- | --- | --- | --- | --- | --- | --- | --- | --- |
| Asymptomatic Genetic Parkinson’s Disease versus Healthy Controls | | | | | | | | |  |
| UPSIT | – |  |  |  |  |  |  |  |  |
| HVLT | 0.21** | – |  |  |  |  |  |  |  |
| Age | -0.09* | -0.25** | – |  |  |  |  |  |  |
| Diagnosis | -0.15* | -0.16** | -0.04 | – |  |  |  |  |  |
| Sex | -0.11* | -0.23** | 0.12** | 0.57** | – |  |  |  |  |
| MoCA | 0.15** | 0.39** | -0.13** | -0.30** | -0.04 | _ |  |  |  |
| Visu-Exec | 0.10* | 0.07 | -0.03 | -0.12** | 0.02 | 0.20** | _ |  |  |
| Delayed-Recall | -0.01 | -0.01 | -0.01 | 0.03 | 0.04 | 0.01 | -0.03 | - |  |
| Attention | 0.06 | 0.09 | 0.05 | -0.11* | 0.03 | 0.15** | 0.20** | 0.07 |  |
| Symptomatic Genetic Parkinson’s Disease versus Healthy Controls | | | | | | | | |  |
| UPSIT | – |  |  |  |  |  |  |  |  |
| HVLT | 0.28** | – |  |  |  |  |  |  |  |
| Age | 0.05 | -0.14** | – |  |  |  |  |  |  |
| Diagnosis | -0.65** | -0.19** | -0.14** | – |  |  |  |  |  |
| Sex | -0.00 | -0.02 | 0.02 | -0.16** | – |  |  |  |  |
| MoCA | 0.36** | 0.53** | -0.08 | -0.40** | 0.06 | – |  |  |  |
| Visu-Exec | 0.21** | 0.20** | -0.06 | -0.25** | 0.02 | 0.42** | _ |  |  |
| Delayed-Recall | 0.18** | 0.33** | -0.05 | -0.25** | 0.04 | 0.48** | 0.22** | _ |  |
| Attention | 0.20** | 0.23** | 0.08 | -0.28** | 0.10* | 0.53** | 0.39** | 0.26** |  |
| Possible Prodromal PD versus Healthy Controls | | | | | | | | |  |
| UPSIT | – |  |  |  |  |  |  |  |  |
| HVLT | 0.36** | – |  |  |  |  |  |  |  |
| Age | -0.25** | -0.26** | – |  |  |  |  |  |  |
| Diagnosis | -0.69** | -0.35** | 0.20** | – |  |  |  |  |  |
| Sex | -0.16** | -0.23** | 0.10 | 0.33** | – |  |  |  |  |
| MoCA | 0.25** | 0.42** | -0.19** | -0.40** | -0.05 | _ |  |  |  |
| Visu-Exec | 0.35** | 0.17** | -0.13* | -0.25** | -0.01 | 0.28** | _ |  |  |
| Delayed-Recall | 0.18** | 0.28** | -0.13* | -0.26** | 0.13* | 0.36** | 0.18** |  |  |
| Attention | 0.26** | 0.12 | -0.08 | -0.24** | -0.02 | 0.20** | 0.53** |  |  |
| Sporadic Parkinson’s Disease versus Healthy Controls | | | | | | |  |  | |
| UPSIT | – |  |  |  |  |  |  |  |  |
| HVLT | 0.24** | – |  |  |  |  |  |  |  |
| Age | -0.22** | -0.28** | – |  |  |  |  |  |  |
| Diagnosis | -0.53** | -0.15** | 0.02 | – |  |  |  |  |  |
| Sex | -0.14** | -0.22** | 0.08* | 0.01 | – |  |  |  |  |
| MoCA | 0.25** | 0.37** | -0.20** | -0.23** | -0.10** | – |  |  |  |
| Visu-Exec | 0.17** | 0.27** | -0.15** | -0.10** | 0.04 | 0.48** | _ |  |  |
| Delayed-Recall | 0.22** | 0.30** | -0.19** | -0.18** | -0.15** | 0.76** | 0.09* | _ |  |
| Attention | 0.06 | 0.13** | -0.03 | -0.12** | 0.04 | 0.49** | 0.12** | 0.19** |  |

**p* < 0.05, ***p* < 0.01. UPSIT: University of Pennsylvania Smell Identification Test. HVLT: Hopkins Verbal Learning Test. MoCA: Montreal Cognitive Assessment Test. Visu-Exec: MoCA subscore for visuospatial and executive function. Delayed-Recall: MoCA subscore for delayed recall. PPMI-defined diagnostic groups: Asymptomatic-genetic-Parkinson’s-disease subjects have a mutation, or are a first-degree relative of an individual having a mutation, in *LRRK2*, *SNCA*, or *GBA*; Symptomatic-genetic-Parkinson’s-disease subjects have a mutation in *LRRK2*, *SNCA*, or *GBA*; Possible-prodromal-PD subjects have REM-behavior sleep disorder and/or hyposmia.
