# Supplementary material for: Cognitive processes that indirectly affect olfactory dysfunction in Parkinson's disease
Source: Clin Park Relat Disord. 2019 Jul 20;1:13–20. doi: 10.1016/j.prdoa.2019.07.003 (PMC8288748; doi:10.1016/j.prdoa.2019.07.003)
Supplement: Supplemental Table 2 — Regression model of UPSIT scores on diagnostic categories, sex, age and MoCA scores. [file mmc4.docx]

**Supplemental Table 2.** Regression model of UPSIT scores on diagnostic categories, sex, age and MoCA scores.

| **Model** | ***b*** | ***SE*** | ***β*** | ***F*** | ***t*** | ***R^2^*** | ***p*** |
| --- | --- | --- | --- | --- | --- | --- | --- |
| **Intercept** | 37.7 | 3.1 |  | 120.6 |  | 0.4 | 4.2 × 10^-32^ |
| **Age** | -0.1 | 0.02 | -0.1 |  | -5.1 |  | 4.8 × 10^-7^ |
| **Sex** | -2.0 | 0.4 | -0.1 |  | -4.8 |  | 2 × 10^-6^ |
| **Symptomatic Genetic Parkinson’s Disease** | -12.6 | 0.7 | -0.5 |  | -17.2 |  | 1.8 × 10^-59^ |
| **Asymptomatic Genetic Parkinson’s Disease** | -1.6 | 0.7 | -0.1 |  | -2.3 |  | 0.022 |
| **Sporadic Parkinson’s Disease** | -10.3 | 0.6 | -0.5 |  | -16.9 |  | 1.2 × 10^-57^ |
| **Possible Prodromal Parkinson’s Disease** | -15.4 | 1.1 | -0.4 |  | -14.5 |  | 4.2 × 10^-44^ |
| **MoCA** | 0.3 | 0.1 | 0.1 |  | 3.1 |  | 0.002 |

PPMI-defined diagnostic groups: Asymptomatic-genetic-Parkinson’s-disease subjects have a mutation, or are a first-degree relative of an individual having a mutation, in *LRRK2*, *SNCA*, or *GBA*; Symptomatic-genetic-Parkinson’s-disease subjects have a mutation in *LRRK2*, *SNCA*, or *GBA*; Possible-prodromal-Parkinson’s-disease subjects have REM-behavior sleep disorder and/or hyposmia. MoCA: Montreal Cognitive Assessment Test.
